# Psychosocial outcomes of children with ear infections and hearing problems: a longitudinal study

**DOI:** 10.1186/1471-2431-14-65

**Published:** 2014-03-04

**Authors:** Anthony Hogan, Rebecca L Phillips, Damien Howard, Vasoontara Yiengprugsawan

**Affiliations:** 1 ANZSOG Institute for Governance, University of Canberra, Canberra ACT 2601, Australia; 2 James Cook University, PO Box 793, Nightcliff NT 0814, Australia; 3 National Centre for Epidemiology and Population Health, The Australian National University, Canberra ACT 0200, Australia

**Keywords:** Hearing, Deaf, Disability, Ear infection, Wellbeing, Mental health

## Abstract

**Background:**

There is some evidence of a relationship between psychosocial health and the incidence of ear infections and hearing problems in young children. There is however little longitudinal evidence investigating this relationship. This paper used 6-year prospective longitudinal data to examine the impact of ear infection and hearing problems on psychosocial outcomes in two cohorts of children (one cohort recruited at 0/1 years and the other at 4/5 years).

**Methods:**

Data from the Longitudinal Study of Australian Children (LSAC) were analysed to address the research aim. The LSAC follows two cohorts of children (infants aged 0/1 years – B cohort, n = 4242; and children aged 4/5 years – K cohort, n = 4169) collecting data in 2004, 2006, 2008 and 2010. In B cohort at baseline 3.7% (n = 189) of the sample were reported by their parent to have had an ear infection (excluding hearing problems) and 0.5% (n = 26) were reported by their parent to have hearing problems (excluding ear infections). 6.7% (n = 323) of the K cohort were identified as having had an ear infection and 2.0% (n = 93) to have hearing problems. Psychosocial outcomes were measured using the Strengths and Difficulties Questionnaire. Data were analysed using multivariate analysis of variance and logistic regression, reporting adjusted odds ratio and 95% confidence intervals of the association between reported ear infections (excluding hearing problems)/or hearing problems (excluding ear infections) and psychosocial outcomes.

**Results:**

Children were more likely to have abnormal/borderline psychosocial outcomes at 10/11 years of age if they had been reported to have ongoing ear infections or hearing problems when they were 4/5 years old. When looking at the younger cohort however, poorer psychosocial outcomes were only documented at 6/7 years for children reported to have hearing problems at 0/1 years, not for those who were reported to have ongoing ear infections.

**Conclusion:**

This study adds further evidence that a relationship may exist between repeated ear infections or hearing problems and the long-term psychosocial health of children and provides support for a more systematic investigation of these issues.

## Background

The literature reports that long-term effects may be associated with transient hearing problems (e.g. ear infections, including otitis media) and permanent hearing problems in young children. Transient hearing problems are also known in some circumstances to result in permanent hearing problems [1,2] or auditory processing problems [3], and children with permanent hearing problems are reported to have poorer language skills than their hearing peers [4-6]. A lack of early auditory stimulation is thought to affect neurocognitive processing and result in these poorer outcomes [7]. Of course, these outcomes tend to be associated with children with more severe degrees of hearing problems. It is unsurprising that there is a wide variety of outcomes reported for this cohort given the variability in the nature and frequency of ear disease, world-wide differences in availability and frequency of interventions and societal attitudes [8,9] as well as the wide distribution in the nature of reported hearing problems. Historically, the literature has been primarily concerned with the physical and cognitive outcomes of ear disease and hearing problems. However, it is increasingly recognized that the psychosocial outcomes also merit attention [10-12].

Psychosocial outcomes are concerned with the psychological and social functioning of a child [13]. A number of cross-sectional studies have investigated the psychosocial outcomes of children with otitis media and hearing problems and predominantly report lower psychosocial outcomes when compared to children without these conditions. In these studies it has been shown that children with hearing problems are estimated to be 3.7 times more likely to have psychosocial difficulties [13,14] and 2-3 times more likely to have moderate to severe mental health problems [15] than hearing children. Specifically, children with hearing problems aged 1.5 -19 years have been reported to have difficulties with: attention [4]; behaviour [4,6,16]; communication [4]; conduct [17]; relationships [17,18]; emotions [17]; and social behaviour [6,19]. It may be assumed that children with more severe hearing problems will have poorer outcomes but it has been reported that children with milder hearing problems actually exhibit the worst psychosocial health related quality of life and behaviour scores [6]. When looking at the impact of ear infections on psychosocial outcomes, children with otitis media aged 0-18 years have been reported to be hyperactive [20-25] and have emotional and behavioural problems [20-23,26].

In contrast, several studies have found no difference in psychosocial outcomes between children with and without hearing problems. A Swedish study involving adolescents aged 11-18 years found no significant difference between children with and without hearing problems [27]. Another study found that health related quality of life was lower than the norm for children with hearing problems aged 8-12 years, but was the same for those aged 13-16 years [28].

Viewed collectively these findings suggest that children with ear infections and hearing problems are likely to have poorer psychosocial outcomes. Longitudinal studies however are required to track psychosocial outcomes and report how they may change over time. Several longitudinal studies have been completed showing mixed results as to the impact of otitis media and hearing problems on long-term psychosocial outcomes. One longitudinal study, in which children were recruited from child care centres, reported that there was no relationship in the first six years of life [29]. In contrast, a population based longitudinal study in New Zealand in the 1970s documented that teachers, but not the parents, reported more behaviour problems across the study period for children with otitis media compared to children without this condition [30]. A strength of this study is its use of a population based sample, which is important for avoiding biases such as selection bias and loss to follow-up [6]. However, this study was completed over 30 years ago and may not reflect the outcomes that children in Australia experience today given the advancement in knowledge and practice over this period.

Contemporary population based longitudinal studies can monitor the psychosocial outcomes of children with a variety of conditions including ear infections and hearing problems over time. These data can be used to investigate whether poorer outcomes are present at certain ages and, if required, guide the development of services and strategies to minimise the long-term impacts of these conditions. This paper therefore uses an existing national dataset to examine the impact of ear infection and hearing problems on psychosocial outcomes over time using 6-year prospective longitudinal data in two cohorts of children (one cohort recruited at 0/1 years and the other at 4/5 years).

## Method

### The Longitudinal Study of Australian Children

This study used data from the Longitudinal Study of Australian Children (LSAC) to undertake longitudinal analysis of the impact of ear infections and hearing problems on the psychosocial outcomes of children. The LSAC follows two cohorts of children (infants aged 0/1 years and children aged 4/5 years) collecting data every two years on the experiences of: children within their families and communities; their health; child care experiences; and their early years of education [31]. Data were collected predominantly from the biological mother (over 97% for both cohorts), as well as from fathers, teachers, carers and direct observation. A two-stage clustered sample design, stratified by state and by metropolitan/urban status was used to randomly select children using the Medicare database [32]. The LSAC study has previously been described in further depth [31,32].

The two cohorts aged 0/1 years (B cohort) and 4/5 years (K cohort) were recruited in 2004 (Wave 1). Waves of interviews have been conducted every two years: 2006 (Wave 2), 2008 (Wave 3) and 2010 (Wave 4). The LSAC cohorts are broadly representative of the Australian population, although there is some over-representation of children with more highly educated parents, as well as under-representation of children from single-parent and non-English speaking families, and families living in rental properties [32,33].

The author has obtained a license to use the LSAC data from the Australian Government Department of Families, Housing, Community Services and Indigenous Affairs.

### Sample

For this study all four waves of data were used for the B and K cohorts. At baseline the B cohort (aged 3-19 months) included a sample of 5,107 children and the K cohort (aged 4 years 3 months to 5 years 7 months) included 4,983 children. At Wave 4 the B cohort included 4,242 children and the K cohort included 4,169 children. The characteristics of the two cohorts have previously been documented by Yiengprugsawan et al. [2] and are presented again in Table 1.

**Table 1:** Characteristics of the B and K cohort

**Characteristics**	**Percent (n)**	
	**B cohort 0/1 years**	**K cohort 4/5 years**
	**N = 4,242**	**N = 4,169**
Child characteristics		
Female	48.9 (2,497)	49.1 (2,446)
English as main language at home	89.2 (4,555)	3.8 (187)
Parent characteristics		
Female	98.6 (5,033)	97.1 (4,839)
Mean age (±*SD*)	31.0 (±5.5)	34.7 (±5.5)
Parent’s employment status		
Employed	49.7 (2,531)	57.4 (2,852)
Unemployed	3.2 (165)	3.8 (188)
Not in labour force	47.1 (2,400)	38.9 (1,932)
Parent’s highest level of school completion		
Year 12+	66.7 (3,404)	58.1 (2,895)
Year 10/11	28.3 (1,443)	34.9 (1,739)
Year 9 or less	5.0 (256)	6.9 (344)
Remoteness area		
Highly accessible	54.8 (2,800)	53.8 (2,655)
Accessible	23.3 (1,188)	23.5 (1,159)
Moderately accessible	16.5 (840)	17.3 (855)
Remote/very remote	4.3 (221)	4.4 (217)
SEIFA economic resources		
Below median	47.3 (2,413)	45.8 (2,284)

### Measures

#### ***Ear infection and hearing problems***

The presence of ear disease in the LSAC was recorded using a categorical question. The responding parent was asked: Does (child of interest) have any of these ongoing conditions – ear infections (yes/no)? Hearing problems were reported by the parent being asked: Does (child of interest) have any of these ongoing conditions – hearing problems (yes/no)? It is not possible with this level of data to determine the nature, duration, severity or repetitiveness of ear infections or hearing problems. Children who were identified as having ear infections (excluding hearing problems) or hearing problems (excluding ear infections) at baseline (Wave 1) were included in the analyses reported in this paper.

#### ***Child emotional and behavioural difficulties***

The Strengths and Difficulties Questionnaire (SDQ, UK version) [34] assesses symptoms of children’s’ emotional distress (e.g. ‘Often unhappy, downhearted or tearful’), conduct and oppositional behaviours (e.g. ‘Often has temper tantrums or hot tempers’), hyperactivity and inattention (e.g. ‘Restless, overactive, cannot stay still for long’) and peer problems (e.g. ‘Picked on or bullied by other children’) (Cronbach’s alpha mothers = 0.79, fathers = 0.79). An overall child difficulties score can be formed by summing the 20 items (response categories 0 = not true, 1 = somewhat true and 2 = certainly true). The SDQ has been found to provide high specificity (94.6%) and reasonable sensitivity (63.3%) in detecting psychiatric disorders. Sensitivity is strongly increased when the child’s wellbeing is rated by multiple raters: ‘the questionnaires identified over 70% of individuals with conduct, hyperactivity, depressive and some anxiety disorders’ [35], p534. Scores can be grouped into the categories normal, borderline and abnormal. In the LSAC, SDQ data were provided by the primary caregiver (mostly mothers), and for the subset at school the child’s teacher. SDQ scores are available for all waves of the K cohort (Waves 1-4), but only from age 4/5 years for the B cohort (Waves 3 and 4).

### Data analysis

Data were analysed using multivariate analysis of variance (MANOVA) and logistic regression. Adjustments were made to account for differences associated with the responding parent’s education and Socio-Economic Indexes for Areas (SEIFA) which ranks area economic resources [36]. MANOVA was first used to determine whether there were significant differences between the psychosocial outcomes of children with hearing problems (excluding ear infections)/ or ear infections (excluding hearing problems) and those children without these conditions. Logistic regression then estimated the adjusted odds ratio and 95% confidence intervals of the association between reported ear infections (excluding hearing problems)/or hearing problems (excluding ear infections) and psychosocial outcomes. Analyses were completed using Stata version 12. Those identified with ear infections or hearing problems at baseline were followed longitudinally.

## Results

In B cohort at baseline 3.7% (n = 189) of the sample was reported to have had an ear infection (excluding hearing problems) and 0.5% (n = 26) were reported to have hearing problems (excluding ear infections). 6.7% (n = 323) of the K cohort were identified as having had an ear infection and 2.0% (n = 93) to have hearing problems. MANOVA revealed that for the B cohort there were no statistically significant differences when comparing the SDQ subscale scores of children with hearing problems (excluding ear infections)/or ear infections (excluding hearing problems) with those of children without these conditions (see Table 2). However, when looking at K cohort, children with hearing problems (excluding ear infections)/or ear infections (excluding hearing problems) had significantly lower SDQ subscale scores than children without these conditions (see Table 3).

**Table 2:** B cohort: multivariate analysis of variance (MANOVA) of psychosocial outcomes of children reporting ear infections or hearing problems

**Psychosocial outcomes**	**MANOVA of Strengths and Difficulties Questionnaire (SDQ) subscales**			
	**Reported ear infections at Wave 1 (0/1 year)**		**Reported hearing problems at Wave 1 (0/1 year)**	
	**SDQ Wave 3 4/5 years**	**SDQ Wave 4 6/7 years**	**SDQ Wave 3 4/5 years**	**SDQ Wave 4 6/7 years**
Wilks’ lambda	0.999, p = 0.635	0.999, p = 0.888	0.999, p = 0.156	0.999, p = 0.751
Pillai’s trace	0.001, p = 0.635	0.001, p = 0.888	0.001, p = 0.156	0.001, p = 0.751
Hotelling trace	0.001, p = 0.635	0.001, p = 0.888	0.001, p = 0.156	0.001, p = 0.751
Roy’s largest root	0.001, p = 0.635	0.001, p = 0.888	0.001, p = 0.156	0.001, p = 0.751

*Note*. Analyses were adjusted for parent’s education and Socio-Economic Indexes for Areas (SEIFA).

**Table 3:** K cohort: multivariate analysis of variance (MANOVA) of psychosocial outcomes of children reporting ear infections or hearing problems

**Psychosocial outcomes**	**MANOVA of Strengths and Difficulties Questionnaire (SDQ) subscales by ear infections or hearing problems**							
	**Reported ear infections at baseline Wave 1 (4/5 years)**				**Reported hearing problems at baseline Wave 1 (4/5 years)**			
	**SDQ Wave 1 4/5 years**	**SDQ Wave 2 6/7 years**	**SDQ Wave 3 8/9 years**	**SDQ Wave 4 10/11 years**	**SDQ Wave 1 4/5 years**	**SDQ Wave 2 6/7 years**	**SDQ Wave 3 8/9 years**	**SDQ Wave 4 10/11 years**
Wilks’ lambda	0.987,	0.993,	0.992,	0.992,	0.991,	0.993,	0.986,	0.991,
	p <0.001	p <0.001	p <0.001	p <0.001	p <0.001	p <0.001	p <0.001	p <0.001
Pillai’s trace	0.013,	0.007,	0.008,	0.008,	0.009,	0.007,	0.014,	0.009,
	p <0.001	p <0.001	p <0.001	p <0.001	p <0.001	p <0.001	p <0.001	p <0.001
Hotelling trace	0.013,	0.007,	0.008,	0.008,	0.009,	0.007,	0.015,	0.009,
	p <0.001	p <0.001	p <0.001	p <0.001	p <0.001	p <0.001	p <0.001	p <0.001
Roy’s largest root	0.013,	0.007,	0.008,	0.008,	0.009,	0.007,	0.015,	0.009,
	p <0.001	p <0.001	p <0.001	p <0.001	p <0.001	p <0.001	p <0.001	p <0.001

*Note*. Analyses were adjusted for parent’s education and Socio-Economic Indexes for Areas (SEIFA).

Table 4 provides data on the longitudinal psychosocial outcomes of children reported to have ear infections (excluding hearing problems) and hearing problems (excluding ear infections) at baseline for B cohort. SDQ data were not collected for Waves 1 and 2 of B cohort, therefore data are only presented for Waves 3 and 4. Findings indicate that abnormal/borderline pro-social and emotional scores at Wave 4 are associated with reporting hearing problems at 0/1 years of age (adjusted odds ratios [AORs] 2.67 and 2.20 respectively).

**Table 4:** B cohort: longitudinal psychosocial outcomes of children reported to have ear infections or hearing problems

**SDQ subscale**	**Baseline ear infections**				**Baseline hearing problems**			
	**SDQ Wave 3 4/5 years**		**SDQ Wave 4 6/7 years**		**SDQ Wave 3 4/5 years**		**SDQ Wave 4 6/7 years**	
	**%**	**AOR**	**%**	**AOR [95% CI]**	**%**	**AOR [95% CI]**	**%**	**AOR [95% CI]**
Abnormal/borderline pro-social score	13.8	1.03, p = 0.846	8.7	1.06, p = 0.724	18.8	1.85, p = 0.122	26.3	2.67*, p = 0.015
		Wald = 0.0, SE = 0.2		Wald = 0.1, SE = 0.2		Wald = 2.4, SE = 0.7		Wald = 5.9, SE = 1.1
Abnormal/borderline hyperactivity score	17.4	1.13, p = 0.429	18.5	1.03, p = 0.832	12.5	1.53, p = 0.292	21.0	1.48, p = 0.333
				Wald = 0.1, SE = 0.2		Wald = 1.1, SE = 0.6		Wald = 0.9, SE = 0.6
		Wald = 0.6, SE = 0.2						
Abnormal/borderline emotional score	10.2	0.99, p = 0.967	19.9	1.19, p = 0.274	12.5	1.73, p = 0.173	31.5	2.20*, p = 0.048
		Wald = 0.0, SE = 0.2		Wald = 1.2, SE = 0.2		Wald = 1.8, SE = 0.7		Wald = 3.9, SE = 0.5
Abnormal/borderline peer problems score	18.9	0.95, p = 0.759	21.9	1.08, p = 0.633	25.0	1.72, p = 0.176	26.3	1.60, p = 0.242
		Wald = 0.1, SE = 0.1		Wald = 0.2, SE = 0.1		Wald = 1.8, SE = 0.7		Wald = 1.4, SE = 0.6
Abnormal/borderline conduct score	35.5	0.89, p = 0.449	25.2	1.13, p = 0.426	50.0	1.90, p = 0.136	21.0	1.26, p = 0.566
		Wald = 0.6, SE = 0.2		Wald = 0.6, SE = 0.2		Wald = 2.2, SE = 0.8		Wald = 0.3, SE = 0.5
Abnormal/borderline total score	15.2	1.03, p = 0.836	19.2	1.22, p = 0.221	25.0	2.09, p = 0.066	21.1	1.69, p = 0.196
		Wald = 0.1, SE = 0.2		Wald = 1.5, SE = 0.2		Wald = 3.4, SE = 0.8		Wald = 1.6, SE = 0.7

*Note.* * = significant difference; AOR = adjusted odds ratio; SE = standard error; SDQ = Strengths and Difficulties Questionnaire. Analyses were adjusted for the responding parent’s education and Socio-Economic Indexes for Areas (SEIFA).

Longitudinal associations between psychosocial outcomes and reported ear infections (excluding hearing problems) or hearing problems (excluding ear infections) at baseline for the K cohort are reported in Table 5. At baseline those with ear infections were more likely to have abnormal/borderline total SDQ scores (AOR = 2.07) and on all SDQ subscales except for pro-social behaviour (AOR: hyperactivity = 1.36, emotional = 2.32, peer problems = 1.43 and conduct = 1.39). However, 6 years later this group of children were no more likely to have an abnormal/borderline total SDQ score than children who were not reported to have ear infections at Wave 1. They were still more likely to have abnormal/borderline SDQ scores for the emotional (AOR = 1.44) and peer problems (AOR = 1.34) subscales.

**Table 5:** K cohort: longitudinal psychosocial outcomes of children reported to have ear infections or hearing problems

**SDQ subscale**	**Baseline ear infections**								**Baseline hearing problems**							
	**SDQ Wave 1 4/5 years**		**SDQ Wave 2 6/7 years**		**SDQ Wave 3 8/9 years**		**SDQ Wave 4 10/11 years**		**SDQ Wave 1 4/5 years**		**SDQ Wave 2 6/7 years**		**SDQ Wave 3 8/9 years**		**SDQ Wave 4 10/11 years**	
	**%**	**AOR**	**%**	**AOR**	**%**	**AOR**	**%**	**AOR**	**%**	**AOR**	**%**	**AOR**	**%**	**AOR**	**%**	**AOR**
Abnormal/borderline pro-social score	13.2	1.13	12.5	1.23	12.1	1.06	10.6	1.21	19.3	1.62	14.7	1.67*	22.8	1.50	13.3	1.32
		p = 0.477		p = 0.129		p = 0.666		p = 0.158		p = 0.072		p = 0.025		p = 0.060		p = 0.234
		Wald = 0.5		Wald = 2.3		Wald = 0.2		Wald = 2.0		Wald = 2.3		Wald = 5.0		Wald = 3.5		Wald = 1.42
		SE = 0.2		SE = 0.2		SE = 0.1		SE = 0.2		SE = 0.4		SE = 0.4		SE = 0.3		SE = 0.3
Abnormal/borderline hyperactivity score	22.8	1.36*	22.1	1.34*	17.8	1.05	19.4	1.17	32.3	2.00*	28.0	1.91*	32.8	1.70*	24.0	1.34
		p = 0.026		p = 0.020		p = 0.678		p = 0.207		p = 0.002		p = 0.003		p = 0.013		p = 0.186
		Wald = 5.0		Wald = 5.4		Wald = 0.2		Wald = 1.6		Wald = 9.3		Wald = 8.9		Wald = 6.1		Wald = 1.7
		SE = 0.2		SE = 0.2		SE = 0.1		SE = 0.1		SE = 0.4		SE = 0.4		SE = 0.4		SE = 0.3
Abnormal/borderline emotional score	26.3	2.32*	21.0	1.40*	21.4	1.26	28.4	1.44*	29.0	2.39*	21.6	1.76*	28.6	1.56*	30.7	1.51
		p = 0.000		p = 0.007		p = 0.056		p = 0.002		p = 0.000		p = 0.010		p = 0.038		p = 0.058
		Wald = 39.1		Wald = 7.2		Wald = 3.7		Wald = 9.3		Wald = 13		Wald = 6.7		Wald = 4.3		Wald = 3.6
		SE = 0.3		SE = 0.2		SE = 0.2		SE = 0.2		SE = 0.6		SE = 0.4		SE = 0.3		SE = 0.3
Abnormal/borderline peer problems score	32.8	1.43*	28.9	1.27*,	33.1	1.34*	30.0	1.33*	40.9	1.83*	30.2	1.46	38.6	1.55*	42.6	1.96*
		p = 0.004		p = 0.044		p = 0.013		p = 0.018		p = 0.005		p = 0.081		p = 0.042		p = 0.002
		Wald = 8.1		Wald = 4.1		Wald = 3.2		Wald = 5.6		Wald = 7.8		Wald = 3.0		Wald = 4.1		Wald = 9.8
		SE = 0.2		SE = 0.2		SE = 0.2		SE = 0.2		SE = 0.4		SE = 0.3		SE = 0.3		SE = 0.4
Abnormal/borderline conduct score	51.9	1.39*	25.0	1.25	22.6	1.13	23.9	1.26	57.0	1.59*	33.3	1.90*	31.4	1.47	38.7	2.00*
		p = 0.005		p = 0.067		p = 0.298		p = 0.056		p = 0.001		p = 0.003		p = 0.076		p = 0.001
		Wald = 8.0		Wald = 3.4		Wald = 1.1		Wald = 3.6		Wald = 4.6		Wald = 9.0		Wald = 3.1		Wald = 10.3
		SE = 0.2		SE = 0.1		SE = 0.1		SE = 0.1		SE = 0.3		SE = 0.4		SE = 0.3		SE = 0.4
Abnormal/borderline total score	32.8	2.07*	21.4	1.42	18.1	1.12	23.9	1.41*	40.8	2.62*	20.3	1.64*	31.5	1.69*	29.3	1.63*
		p = 0.000		p = 0.006		p = 0.349		p = 0.005		p < 0.001		p = 0.026		p = 0.015		p = 0.025
		Wald = 33.0		Wald = 7.6		Wald = 0.9		Wald = 7.9		Wald = 19		Wald = 4.9		Wald = 5.9		Wald = 5.0
		SE = 0.3		SE = 0.2		SE = 0.1		SE = 0.2		SE = 0.6		SE = 0.4		SE = 0.4		SE = 0.2

*Note.* * = significant difference; AOR = adjusted odds ratio; SE = standard error; SDQ = Strengths and Difficulties Questionnaire. Analyses were adjusted for the responding parent’s education and Socio-Economic Indexes for Areas (SEIFA).

Children who were reported to have hearing problems at baseline (4/5 years of age) were more likely to have abnormal/borderline total SDQ scores across all four waves. At Waves 2 and 3, they were more likely to have abnormal/borderline scores for four of the five subscales. However, at 10/11 years of age (Wave 4) they were only more likely to have these scores on two of the subscales (AOR: peer problems = 1.96 and conduct = 2.00).

## Discussion

By using longitudinal data this paper examined the impact of ear infections and hearing problems on children’s long-term psychosocial outcomes and provided preliminary evidence that a relationship may exist between the former and the latter. Children were more likely to have abnormal/borderline psychosocial outcomes at 10/11 years of age if they had been reported to have ongoing ear infections or hearing problems when they were 4/5 years old. When looking at the younger cohort however, poorer psychosocial outcomes were only documented at 6/7 years for children reported to have hearing problems at 0/1 years, not for those who were reported to have ongoing ear infections.

The findings suggest that the older cohort of children had poorer psychosocial outcomes than the younger cohort. There are several possible reasons for this discrepancy. Ongoing ear infections at 0/1 years of age may not necessarily result in long-term psychosocial problems because these children may not continue to have ear infections, potentially reducing their impact on the child’s psychosocial development. In comparison, children aged 4/5 years are in a key developmental period [37] and ongoing ear infections may have already and may continue to impact on their ability to develop language and literacy skills, as well as to learn and be included within group situations [38]. The findings may also in part be explained by parents being more accurate in identifying that their child has ongoing ear infections when they are aged 4/5 years rather than 0/1 years; therefore some children with ear disease may have been excluded from the 0/1 year old cohort. It is argued that the accuracy of parent-report of ear infection is low in children aged under two years [39] but it is not known if this accuracy increases as the child becomes older. Ear disease is often asymptomatic and/or relies on children communicating that they have sore ears, which children may be more able to do at 4/5 years of age.

Although children who were reported to have hearing problems at 0/1 years and 4/5 years both had poorer psychosocial outcomes six years later, the impact was more marked in the older cohort who received an abnormal/borderline total SDQ score, in addition to abnormal/borderline for two of the subscales (which both cohorts received). When examining the two cohorts in further depth (by comparing them when they were both 6/7 years) it is apparent that there are differences between the groups. Children who were reported to have hearing problems at 0/1 years of age were more likely to have abnormal/borderline scores for two subscales (pro-social and emotional behaviour) when they reached 6/7 years of age. In comparison, children who were reported to have ongoing hearing problems at age 4/5 years were more likely to have abnormal/borderline scores for four out of the five subscales, as well as for the total score, when they were 6/7 years of age. There are a number of possible explanations for these outcomes. This result suggests that hearing problems present at 0/1 years of age result in fewer psychosocial problems at 6/7 years than those who have hearing problems at 4/5 years of age. This could be because what is thought to be hearing problems at 0/1 years resolves; a result in part of earlier, more effective and historically more recent interventions. Alternately, the 4/5 year age group captures those children with long-term hearing problems or such problems may only manifest in a more serious way, after many years of repeated problems. Taken together with the results of other longitudinal work, which suggests that the prevalence of such problems is much higher than first thought [2], this research suggests that the broader impact of ear disease and hearing problems in children may have been under-estimated and as such warrants further research.

This argument is underscored by the fact that children with milder hearing problems have been reported to exhibit the worst psychosocial health related quality of life and behaviour scores [6]. These children may not have language delays or receive ongoing intervention but they may experience persistent, seemingly minor communication problems, particularly in noisy settings such as classrooms and the home. These communication breakdowns, although minor in their appearance, have been described by Hétu and Getty [40] as being frequently misperceived as relational conflict. Such relational conflict is likely to have some impact on the poorer psychosocial outcomes illuminated in this study. Consideration is required as to how the current approach to managing ear infections and hearing problems in young children can be extended to address psychosocial needs.

A strength of this paper is the use of a nationally representative longitudinal study that follows two cohorts of children. However, the analysis is limited because it is reliant on parent report of ongoing ear infections or hearing problems, data related to the type or degree of impairment were not collected and it is not known what amplification or treatment a child may have received. Relying on parent report may result in some children who do not have ear infections or hearing problems being included in the sample. For example, parents may initially report that a child has hearing problems but later the child may be identified as having attention deficit hyperactive disorder or autism spectrum disorder instead (the parents may have initially interpreted the symptoms of these conditions as the child not hearing properly). Nevertheless, the findings in this paper demonstrate that children whose parent’s report that they have hearing problems (whether or not they are later diagnosed with a hearing problem) are at risk of poorer psychosocial health, which should not be overlooked by clinicians.

The limitations of this study are partially off-set by the longitudinal nature of these data, the very large protocol fielded and the size of the population sample utilised, making it less likely that parents would consistently provide false reports on these specific items over so many years. Further investigation is warranted to examine the impact that these additional factors have on psychosocial health and strategies for improving the psychosocial outcomes of children with ongoing ear infections and hearing problems.

## Conclusion

This paper contributes to the limited longitudinal evidence available on the psychosocial outcomes of children with ear infections and hearing problems. The findings of this study lend further support to the thesis that a relationship may exist between children who experience ongoing ear infections or have hearing problems and their long-term psychosocial health. Moreover, it provides evidential support for investigating this issue using a more rigorous methodology than has been possible to justify to date. Such insights would in turn lend justification to the development of a deeper understanding of how degree of impairment, communication breakdowns, social inclusion, amplification and treatment received impact on psychosocial wellbeing.

## Competing interests

This study was partially supported by an unconditional grant from GlaxoSmithKline. The authors declare that they have no competing interests.

## Authors’ contributions

AH, DH and VS conceptualised and designed the study. VY analysed the data. AH and RP interpreted the data. RP drafted the manuscript. AH, DH and VY provided comments on the initial manuscript and subsequent revisions. All authors approved the final manuscript.

## Pre-publication history

The pre-publication history for this paper can be accessed here:

http://www.biomedcentral.com/1471-2431/14/65/prepub

## References

[B1] Kong K Coates HL Natural history, definitions, risk factors and burden of otitis media Med J Aust 2009 191 9 Suppl S39 S43 19883355 10.5694/j.1326-5377.2009.tb02925.x PMC7168379

[B2] Yiengprugsawan V Hogan A Strazdins L Longitudinal analysis of ear infection and hearing impairment: findings from 6-year prospective cohorts of Australian children BMC Pediatr 2013 13 28 10.1186/1471-2431-13-28 23432915 PMC3616953

[B3] Williams CJ Jacobs AM The impact of otitis media on cognitive and educational outcomes Med J Aust 2009 191 9 Suppl S69 S72 19883361 10.5694/j.1326-5377.2009.tb02931.x

[B4] Barker DH Quittner AL Fink NE Eisenberg LS Tobey EA Niparko JK Team; TCI Predicting behavior problems in deaf and hearing children: The influences of language, attention and parent-child communication Dev Psychopathol 2009 21 2 373 392 10.1017/S0954579409000212 19338689 PMC2730756

[B5] Sarant J Holt C Dowell R Rickards F Blamey P Spoken language development in oral preschool children with permanent childhood deafness J Deaf Stud Deaf Educ 2008 14 2 205 217 10.1093/deafed/enn034 18840616

[B6] Wake M Hughes EK Poulakis Z Collins C Rickards FW Outcomes of children with mild-profound congenital hearing loss at 7 to 8 years: a population study Ear Hearing 2004 26 1 1 8 14770013 10.1097/01.AUD.0000111262.12219.2F

[B7] Pisoni DB Conway CM Kronenberger WG Horn DL Karpicke J Henning SC Marschark M, Hauser PC Efficacy and effectiveness of cochlear implants in deaf childen Deaf Cognition 2008 Oxford, NY: Oxfort University Press

[B8] Karl A O’Donoghue GM Profound deafness in childhood New Engl J Med 2010 363 15 1438 1450 10.1056/NEJMra0911225 20925546

[B9] Fellinger J Holzinger D Pollard R Mental health of deaf people Lancet 2012 379 9820 1037 1044 10.1016/S0140-6736(11)61143-4 22423884

[B10] Hogan A Shipley M Strazdins L Purcell A Baker E Communication and behavioural disorders among children with hearing loss increases risk of mental health disorders Aust NZ J Publ Heal 2011 35 4 377 383 10.1111/j.1753-6405.2011.00744.x 21806734

[B11] Moeller MP Current state of knowledge: psychosocial development in children with hearing impairment Ear Hearing 2007 28 6 729 739 10.1097/AUD.0b013e318157f033 17982361

[B12] Wake M Hughes EK Collins CM Poulakis Z Parent-reported health-related quality of life in children with congenital hearing loss: a population study Ambul Pediatr 2004 4 5 411 417 10.1367/A03-191R.1 15369416

[B13] Dammeyer J Psychosocial development in a Danish population of children with cochlear implants and deaf and hard-of-hearing children J Deaf Stud Deaf Educ 2009 15 1 50 58 19745003 10.1093/deafed/enp024

[B14] Hintermair M Prevalence of socioemotional problems in deaf and hard of hearing children in Germany Am Ann Deaf 2007 152 3 320 330 10.1353/aad.2007.0028 18018674

[B15] Eldik TV Mental health problems of Dutch youth with hearing loss as shown on the youth self report Am Ann Deaf 2005 150 1 11 16 10.1353/aad.2005.0024 15969220

[B16] Stevenson J McCann D Watkin P Worsfold S Kennedy C The relationship between language development and behaviour problems in children with hearing loss J Child Psychol Psychiatry 2010 51 1 77 83 10.1111/j.1469-7610.2009.02124.x 19686333

[B17] Fellinger J Holzinger D Sattel H Laucht M Mental health and quality of life in deaf pupils Eur Child Adolesc Psychiatry 2008 17 7 414 423 10.1007/s00787-008-0683-y 18810312

[B18] Gilman R Easterbrooks SR Frey M A preliminary study of multidimensional life satisfaction among deaf/hard of hearing youth across environmental settings Soc Indic Res 2004 66 1–2 143 164

[B19] Remine MD Brown M Comparison of the prevalence of mental health problems in deaf and hearing children and adolescents in Australia Aust NZ J Psychiat 2010 44 4 351 357 10.3109/00048670903489866 20307167

[B20] Brouwer CN Rovers MM Maille AR Veenhoven RH Grobbee DE Sanders EA Schilder AG The impact of recurrent acute otitis media on the quality of life of children and their caregivers Clin Otolaryngol 2005 30 3 258 265 10.1111/j.1365-2273.2005.00995.x 16111423

[B21] Brouwer CN Maille AR Rovers MM Grobbee DE Sanders EA Schilder AG Health-related quality of life in children with otitis media Int J Pediatr Otorhinolaryngol 2005 69 8 1031 1041 10.1016/j.ijporl.2005.03.013 16005345

[B22] Timmerman A Meesters C Anteunis L Chenault M Level of psychosocial adaptation in young school children with otitis media Int J Pediatr Otorhinolaryngol 2007 71 12 1843 1848 10.1016/j.ijporl.2007.08.006 17889940

[B23] Gouma P Mallis A Daniilidis V Gouveris H Armenakis N Naxakis S Behavioral trends in young children with conductive hearing loss: a case-control study Eur Arch Otorhinolaryngol 2011 268 1 63 66 10.1007/s00405-010-1346-4 20665042

[B24] Hagerman RJ Falkenstein AR An association between recurrent otitis media in infancy and later hyperactivity Clin Pediatr 1987 26 5 253 257 10.1177/000992288702600508 3568530

[B25] Adesman AR Altshuler LA Lipkin PH Walco GA Otitis media in children with learning disabilities and in children with attention deficit disorder with hyperactivity Pediatr 1990 3 2 442 446 2304807

[B26] Bellussi L Mandala M Passali FM Passali GC Lauriello M Passali D Quality of life and psycho-social development in children with otitis media with effusion Acta Otorhinolaryngol Ital 2005 25 6 359 364 16749604 PMC2639897

[B27] Mejstad L Heiling K Svedin CG Mental health and self-image among deaf and hard of hearing children Am Ann Deaf 2009 153 5 504 515 10.1353/aad.0.0069 19350957

[B28] Huber M Health-related quality of life of Austrian children and adolescents with cochlear implants Int J Pediatr Otorhinolaryngol 2005 69 8 1089 1101 10.1016/j.ijporl.2005.02.018 15946746

[B29] Minter KR Roberts JE Hooper SR Burchinal MR Zeisel SA Early childhood otitis media in relation to children’s attention-related behavior in the first six years of life Pediatr 2001 107 5 1037 1042 10.1542/peds.107.5.1037 11331683

[B30] Silva PA Chalmers E Stewart I Some audiological, psychological, educational and behavioral characteristics of children with bilateral otitis media with effusion: a longitudinal study J Learn Disabil 1986 19 3 165 169 10.1177/002221948601900307 3958605

[B31] Sanson A Nicholson J Ungerer J Zubrick S Wilson K Ainley J Berhelsen D Bittman M Broom D Harrison L Rodgers B Sawyer M Silburn S Strazdins L Vimpani G Wake M Introducing the Longitudinal Study of Australian Children, LSAC Discussion Paper No. 1 2002 Melbourne: Australian Institute of Family Studies

[B32] Soloff C Lawrence D Johnstone R Sample design, LSAC Technical Paper No. 1 2005 Australian Institute of Family Studies: Melbourne

[B33] Soloff C Lawrence D Mission S Johnstone R Wave 1 weighting and non-response, LSAC Technical Paper No. 3 2006 Australian Institute of Family Studies: Melbourne

[B34] Goodman R The strengths and difficulties questionnaire: a research note J Child Psychol Psychiatry 1997 38 5 581 586 10.1111/j.1469-7610.1997.tb01545.x 9255702

[B35] Goodman R Ford T Simmons H Gatward R Meltzer H Using the Strengths and Difficulties Questionnaire (SDQ) to screen for child psychiatric disorders in a community sample Br J Psychiatry 2000 177 534 539 10.1192/bjp.177.6.534 11102329

[B36] ABS Information paper: An introduction to Socio-Economic Indexes for Areas (SEIFA), ABS Catalogue No. 2039.0 2006 Canberra: Australian Bureau of Statistics

[B37] Santrock JW Child development 2009 12 New York, NY: McGraw-Hill

[B38] Winskel H The effects of an early history of otitis media on children’s language and literacy skill development Brit J Educ Psychol 2006 76 727 744 10.1348/000709905X68312 17094883

[B39] Anteunis LJC Engel JAM Hendriks JJT Mamni JJ A longitudinal study of the validity of parental reporting in the detection of otitis media and related hearing impairment in infancy 1999 38 75 82 10.3109/00206099909073006 10206516

[B40] Hétu R Getty L Development of a rehabilitation program for people affected with occupational hearing loss 1: a new paradigm Int J Audiol 1991 30 6 305 316 10.3109/00206099109072893 1772381

